# Validation of the Canadian French version of the fear of COVID‐19 scale in the general population of Quebec

**DOI:** 10.1002/brb3.2550

**Published:** 2022-03-30

**Authors:** Randa Attieh, Kouamé Koffi, Moustapha Touré, Érica Parr‐Labbé, Amir H. Pakpour, Thomas G. Poder

**Affiliations:** ^1^ Department of Management Evaluation and Health Policy School of Public Health University of Montreal Montreal Canada; ^2^ Département de Santé Publique UFR Sciences Pharmaceutiques Universite Felix Houphouet‐Boigny Abidjan, Côte d'Ivoire Canada; ^3^ Department of Economics Scool of Management University of Sherbrooke Sherbrooke Canada; ^4^ University of Sherbrooke Sherbrooke Canada; ^5^ Department of Nursing, School of Health and Welfare Jönköping University Gjuterigatan 5 Jönköping Box 1026 551 11 Sweden; ^6^ Centre de Recherche de l'IUSMM CIUSSS de l'Est de l'île de Montréal University of Montreal Montreal Canada

**Keywords:** Canadian French, COVID‐19, fear of COVID‐19 scale, psychometric tests, translation

## Abstract

**Background:**

The purpose of this study was to develop a Canadian French translation of the fear of COVID‐19 scale (FCV‐19S) and assess its psychometric characteristics.

**Methods:**

A forward and backtranslation process was conducted for the Canadian French version of the FCV‐19S. The guidance of the ISPOR task force for translation and cultural adaptation was followed and cognitive debriefing interviews were conducted with six citizens. The final proofread Canadian French FCV‐19S was then administered to a large sample of citizens from the province of Quebec in Canada through an online survey. A quota sampling was conducted in 2020. Respondents from the survey also completed the Clinical Outcomes in Routine Evaluation (CORE)‐6D and the Sense of Coherence (SOC‐3) questionnaires. Several psychometric tests were performed to investigate the reliability (internal consistency) and validity of the Canadian French FCV‐19S, including construct validity, concurrent validity, and Rasch analysis.

**Results:**

The translation process was conducted without any major difficulties. The cognitive debriefing interviews led to no change in the reconciled translation. The survey collected answers from 3428 citizens. Results indicated that the factor structure of the Canadian French FCV‐19S is a unidimensional factor fitting well with the data. The scale showed adequate reliability (Cronbach's alpha of .903) and concurrent validity, as indicated by significantly negative correlation with CORE‐6D (*r* = –.410) and SOC‐3 (*r* = –.233). The Canadian French FCV‐19S properties tested using Rasch analysis was also very satisfactory.

**Conclusions:**

The results of the present study indicated that the Canadian French version of FCV‐19S is a unidimensional tool with robust psychometric properties in the adult's population of all ages residing in the province of Quebec, Canada.

## Abstract 2

This study developed a Canadian French translation of the fear of COVID‐19 scale (FCV‐19S) and assessed its psychometric characteristics.

## 1 BACKGROUND

In April 2020, the 2019 coronavirus pandemic (COVID‐19), according to the World Health Organization, has become a global health emergency affecting 180 countries and territories (WHO, 2020), and generating multiple socioeconomic and psychological consequences in many countries, including Canada (Roy et al., 2020; Tandon, 2020; Xiang et al., 2020). In addition to freezing all educational and business activities for months, the global pandemic, as in other pandemics, has brought fear, anxiety, and worries among the population as major psychological consequences (Roy et al., 2020; Tandon, 2020; Xiang et al., 2020). The uncertainty surrounding coronavirus including its clinical symptoms, treatment, and vaccination is the hardest thing to handle. People still don't know exactly how they'll be impacted, how long this will last, or how bad things might get. And that makes it all too easy to catastrophize and spiral out into overwhelming dread and panic (Ahorsu et al., 2020).

According to Arora et al. (2020), COVID‐19 affects all aspects of life, and the risk factors are more diverse and numerous than in other pandemics. Therefore, fear and its harmful psychosocial consequences, as the literature points out, can go beyond illness or death (Yoon, 2020; Arora et al., 2020). And this by contracting the virus and inducing other fears such as infecting others, development of denial or phobia for infected people (Pappas et al., 2009; Falagas & Kiriaze, 2006), neuroticism in participants who have experienced a loss of a loved one (Murphy & Moret‐Tatay, 2021), economic hardship (Yoon, 2020; Arora et al., 2020), the exacerbation of the infectious disease itself (Ahorsu et al., 2020; Pappas et al., 2009), and the labeling of infected people perceived to be the origin of the disease (Pappas et al., 2009; Falagas & Kiriaze, 2006).<COMP: Please set reference citations as per the journal style, that is, in alphabetical order.>

Attitudes are also of interest in understanding the effects of COVID‐19 outbreak. Sense of belonging to the country of reference has been shown to be related to and directly predict fear (Murphy & Moret‐Tatay, 2021). Additionally, the protective measures such as lockdowns and quarantines associated with the pandemic COVID‐19 have been shown to have major effects on stress and fear (Lu et al., 2020; CDC, 2020).

As the fear of encountering individuals who may have been infected has been reported in the context of COVID‐19 (CDC, 2020a, 2020b; Lin et al., 2020), a new psychometric assessment tool assessing an individual's fear of COVID‐19 was recently developed by Ahorsu et al. (2020), that is, the fear of COVID‐19 Scale (FCV‐19S), a short and valid robust assessment scale (Lin et al., 2021), across different populations. This scale has been used in countries such as Iran (Alyami et al., 2021), France (Mailliez et al., 2021), Bangladesh (Sakib et al., 2020), Italy (Soraci et al., 2020), Turkey (Satici et al., 2021), Russia and Belarus (Reznik et al., 2021), Israel (Tzur Bitan et al., 2020), Peru (Huarcaya‐Victoria et al., 2022), and Paraguay (Barrios et al., 2021). In addition, in the province of Quebec, the rising fear of COVID‐19 among nursing staff was measured using the validated French‐Canadian (French‐CA) and English‐Canadian (English‐CA) versions of the fear of COVID‐19 scale (FCV‐19S) (Gélinas et al., 2021). Even though the FCV‐19S was validated in a large sample of nursing staff, additional validation testing with other multidisciplinary members is required to substantiate its validity in the Quebec health care workforce (Gélinas et al., 2021).

Given the degree to which the province of Quebec in Canada has been hit by the spread of COVID‐19, we aimed to develop a Canadian French translation of the fear of COVID‐19 scale (FCV‐19S) in the general population, which was conceptually equivalent to the English version. As French‐Canadian is significantly different from the French spoken in France in addition to cultural differences, the validation of a French‐Canadian version in a Quebec ers sample was warranted in light of the previous tools translated from French‐Canadian to French‐France (Lonjon et al., 2014). The aims of this study were to (i) describe the translation and linguistic validation process; (ii) study the psychometric characteristics of the Canadian French translation of the FCV‐19S utilizing principal component analysis (PCA), concurrent validity, Rasch analysis, and reliability; (iii) determine the FCV‐19S score in a Quebecers sample; and (iv) confirm whether the Canadian French FCV‐19S is unidimensional as it was found in the original validation study by Ahorsu et al. (2020).

## 2 METHODS

### 2.1 Transcultural adaptation and validity

The FCV‐19S, originally developed in Farsi (Ahorsu et al., 2020) and then in English (Winter et al., 2020), was translated from the English version by experienced translators into French. It is a seven‐item scale that assesses the fear of COVID‐19. The seven items (e.g., “I am most afraid of coronavirus‐19”) are rated on a 5‐point scale from 1 (strongly disagree) to 5 (strongly agree) with scores ranging from 7 to 35. The higher the score, the greater the fear of COVID‐19. In this study, we followed the principles indicated by the ISPOR task force for translation and cultural adaptation (Wild et al., 2005).

#### 2.1.1 Forward and backtranslations

Two native French speakers fluent in English initially translated independently the English version of the FCV‐19S into French. One of the translators (TGP) has a PhD in health economics, and the other (KK) a master's in pharmacy. In the forward translation process, careful attention was given to the readability and clarity of the items used. The two forward translations were reconciled into a third French translation by TGP, which was then reviewed by KK for avoidance of ambiguities and discrepancies of words, sentences, and meanings. According to KK comments, the third French translation has been adjusted by TGP. A backtranslation (French to English) of the reconciled French translation was carried out to assure the quality of translation between source and target language (Maneesriwongul & Dixon, 2004). The French FCV‐19S was backtranslated by a health care professional English‐speaker who is fluent in French (EPL) and have a bachelor in psychoeducation. A linguistic equivalence was achieved between the English version of the FCV‐19S and the back translated version by TGP and EPL to produce a final forward translation. The back translated version was also assessed by the developer of the FCV‐19S (AHP) to avoid any misunderstanding and ensure a conceptual equivalence. A linguist translator external to the research team was then asked to review all aspects of the final forward translation, including the choice of terminology and meaning, for validation.

#### 2.1.2 Linguistic validation (cognitive debriefing)

For linguistic validation purpose, individual interviews were conducted with six citizens. All respondents were interviewed in French and asked to answer a series of questions about the meaning of each instruction, question, and response option of the French version of the FCV‐19S. The questions were: What does this item mean to you? (Respondents were asked to rephrase the item using their own words); Are the response options consistent with this item? All responses, suggestions, and comments were summarized. The research team reviewed and discussed the responses. A final proofread version was then produced.

### 2.2 Psychometric validation

#### 2.2.1 Participants and procedure

Participants were invited to take part in the study via an online survey that was conducted by the survey company Dynata inc. Quota sampling was performed in 2020 among the general population according to age, gender, and educational level. Inclusion criteria for volunteers were being (i) at least 18 years old, (ii) French‐speaking citizens, (iii) residents of the province of Quebec, Canada, and (iv) agreeing to participate in the study. All participants completed the survey anonymously and gave their informed online consent. All procedures conducted were approved by our local ethics committee review board at the CIUSSS de l'Est de l’île de Montréal.

#### 2.2.2 Measures

Questions concerning sociodemographic aspects of the participants (e.g., age, gender, educational level, marital status, occupation, income, body mass index [BMI], urban or rural, country of origin, residency status) were included in the online survey. This was done along the use of self‐reported questions on health and life satisfaction, and risk acceptance on a 11‐level scale.

The CORE‐6D scale (Mavranezouli et al., 2013) is a 6‐item health descriptive system consisting of a 5‐item unidimensional emotional component (e.g., I never feel terribly alone and isolated) and a physical item (I am never troubled by aches, pains, or other physical problems). Each item has three levels. The unidimensional emotional component of CORE‐6D, combined with the physical item, creates a 2‐dimensional scale, tapping emotional, and physical symptoms in people with common mental disorders. As many items in the CORE‐6D relate to fear and anxiety, it was considered appropriate to use this instrument to test the concurrent validity of the FCV‐19S.

The Sense of Coherence (SOC‐3) scale (Schumann et al., 2003) is a concept that reflects the ability to cope with stress and is at the core of the salutogenesis theory. It consists of at least three dimensions: the comprehensibility, the manageability, and the meaningfulness components (e.g., Do you usually see a solution to problems and difficulties that other people find hopeless?). The 3‐point SOC‐3 is the simplified 3‐item version, which was used in our study to measure the sense of coherence. The sum score of the SOC‐3 ranged from 0 to 6, and higher values indicated a higher sense of Coherence.

The items of the Canadian French FCV‐19S were independently translated and backtranslated by the research team. Additionally, the Canadian French FCV‐19S was pilot‐tested on six participants of different ages and education levels to investigate if there were any problems in understanding the items themselves. The English version of FCV‐19S has shown to be a sound unidimensional scale with high internal consistency (Winter et al., 2020). It displays sound concurrent validity and can be used with confidence among English‐speaking populations (Winter et al., 2020).

#### 2.2.3 Data analysis

The statistics were analyzed using SPSS V.26 (for descriptive statistics, PCA, concurrent validity) and WINSTEP (for Rasch analysis). Descriptive statistics were used to describe the study participants’ characteristics.

The psychometric properties were first examined as follows: descriptive statistics of the FCV‐ 19S items (i.e., means and confidence interval of the main items); item‐intercorrelations (Spearman's ρ), and corrected item‐total correlations (Spearman's ρ between the respective item and the total score without the respective item). An acceptable value for the corrected item to‐total correlation should be >.4 (Wang et al., 2007).

For reliability of the FCV‐19S, an internal consistency calculation was carried out using Cronbach's alpha which, with a value greater than 0.7 indicates satisfactory internal consistency and good test reliability (Nunnally & Bernstein, 1994; Lin et al., 2018a). Also, a Cronbach's alpha was calculated by excluding one item at a time. If values increased by more than 2%, the item was considered potentially problematic.

Principal component analysis for construct validity was conducted using SPSS V.26 to measure the factor structure of the Canadian French FCV‐19S. The aim of this analysis was to examine the dimensionality of the FCV‐19S (Bagozzi & Yi, 1988; Lin et al., 2019). In this study, eigenvalues 0.8 or higher were used to create a matrix with varimax rotation and obtain one component. The weight attributed to each item helped identify items with a low contribution to the variance. Items with a weight above 0.40 were considered good for a component explaining more than 20% of the total variability. For a component explaining less than 20% of the variability, items were considered good if their contribution was above 0.70.

For concurrent validity, we examined the association of the FCV‐19S with the Sense of Coherence scale 3‐item and the CORE‐6D scale using the Pearson's correlation coefficient (*r*). Correlation coefficients less than .25 were considered as poor correlation; between .25 and .5 as moderate; between .5 and .75 as good; and higher than .75 as excellent correlation.

The following properties of the FCV‐19S were examined using the Rasch model: item statistics, item and person separation reliability, item and person separation index, and differential item functioning (DIF). Rasch analysis is a statistical technique traditionally used for binary data, but some polytomous generalizations can be used for interval data (Lin & Pakpour, 2017). Rasch analysis with Ministep (Winsteps) Rasch was used to assess the unidimensionality and item fits of the Canadian French version of FCV‐19S (Sakib et al., 2020; Lin & Pakpour, 2017). Information‐weighted fit statistic (infit) mean square (MnSq) and outlier‐sensitive fit statistic (outfit) MnSq were generated in the Rasch analysis to indicate the item validity. More specifically, a good fit of an item should have both infit and outfit MnSq between 0.5 and 1.5, where a value lower than 0.5 indicates too much redundancy and a value higher than 1.5 indicates too much misfit (Jafari et al., 2012; Khan et al., 2013). Rasch analysis also generates item and person separation reliability (acceptable value > 0.7; Chang et al., 2014); item and person separation index (acceptable value > 2; Chang et al., 2014). Explaining at least 50% of the variance in the Rasch dimension, and an eigenvalue of less than 2.0 on first contrast, provides evidence of unidimensionality (Linacre, 2012).

## 3 RESULTS

### 3.1 Translation and transcultural validation results

In the reconciliation process of the two forward translations from English to Canadian French, 10 modifications of words or sentences were made for a better sounding in Canadian French. The reconciled forward translation was sent for backtranslation and produced an English version nearly identical to the original English version of the FCV‐19S. No changes were suggested for the final version of the Canadian French translation despite having identified 11 passages with words different from the original English version in the backtranslated version, since the differences were interpreted as having the same meaning. This was confirmed by the developer of the FCV‐19S. See Appendix for the Canadian French version of the FCV‐19S.

#### 3.1.1 Linguistic validation

The linguistic validation was carried out with 6 citizens—3 men and 3 women—aged between 24 and 67 years. Mean age was 42 years (*SD* ± 16.88). Five out of six were employed (83.3%) and one was retired. Four have high educational background with a graduate university diploma and two have secondary education level. All respondents were comfortable with the questionnaire, did not have any difficulty understanding the instructions and the response options made lots of sense for them. The meaning was as expected, and they did not have any suggestions to help improving the questionnaire.

### 3.2 Psychometric validation

A total of 3547 citizens consented to participate in the study and 3428 were included for analysis. A total of 119 were excluded for the following reasons: missing data, aged less than 18, and nonresidents of Quebec. Data collection occurred from July to November 2020 and mean time to complete was around 15 min. Table 1 shows the characteristics of the 3428 participants who completed the Canadian French FCV‐19S. Mean age was 52 years (*SD* ± 15.567) with slightly more than half being female (*n* = 1771; 51.7%). A total of 1220 participants (36%) have shown a healthy BMI ranging between 18.5 and 24.9. Results also indicate that 48% of the participants were workers. Also, it is important to highlight that about 37% (*n* = 1270) of the respondents have achieved secondary education level or less and 35.1% hold a university diploma. The mean annual household income was 65,357 Canadian dollars. All these numbers were very similar to those found in other studies conducted in Quebec and representative of provincial statistics (Poder et al., 2020).

**TABLE 1:** Study sample characteristics (*n* = 3428)

	Frequency	Percentage
**Age (years)**		
18–24	141	4.1
25–29	197	5.7
30–34	262	7.6
35–39	265	7.7
40–44	251	7.3
45–49	264	7.7
50–54	304	8.9
55–59	391	11.3
60–64	418	12.2
65–69	509	14.8
70–74	275	8.0
≥ 75	151	4.4
Mean	52	
**Gender**		
Men	1651	48.2
Women	1771	51.7
Intersex	6	0.2
**Body mass index**		
<18.5	113	3.3
18.5–24.9	1220	35.6
25–29.9	1139	33.2
30–34.9	544	15.9
35–39.9	220	6.4
≥ 40	129	3.8
Missing	63	1.8
**Smoking**		
Yes	658	19.2
No	2770	80.8
**Marital status**		
Single	957	27.9
Married/living with a partner	2009	58.6
Divorced/separated	354	10.3
Widowed	108	3.2
**Occupational status**		
Employed or self employed	1652	48.2
Retired	1256	36.6
At home	157	4.6
Student	101	2.9
Unemployed	152	4.4
Sick and parental leave	110	3.2
**Education**		
Secondary or less	1270	37.0
College	957	27.9
University	1201	35.1
**Annual household income, $CAN**		
<5000	63	1.8
5000–9999	48	1.4
10,000–19,999	264	7.7
20,000–29,999	193	5.6
30,000–39,999	347	10.1
40,000–49,999	388	11.3
50,000–59,999	371	10.8
60,000–69,999	290	8.5
70,000–79,999	275	8.0
80,000–99,999	525	15.3
100,000–119,999	261	7.6
120,000–149,999	219	6.4
≥150,000	184	5.4
**Residential area**		
Rural	1010	29.5
Urban	2418	70.5
**Owning a home**		
Owner	2163	63.1
Tenant	1265	36.9
**Residence permit**		
Temporary residence permit	41	1.2
Permanent residency	86	2.5
Canadian citizen	3301	96.3
**Parents’ origins country** ^a^		
Canada	3038	88.6
France	108	3.2
Algeria	25	0.7
Others	257	7.5
**Origin's country** ^a^		
Canada	3124	91.1
France	92	2.7
Algeria	22	0.6
Others	190	5.5
**Health status**		
Excellent	374	10.9
Very good	1324	38.6
Good	1321	38.5
Fair	337	9.8
Bad	72	2.1
**Financial losses due to COVID‐19 pandemic**		
No financial loss	1638	47.8
Small financial losses	1240	36.2
Fairly significant financial losses	466	13.6
Very significant financial losses	84	2.5
**Health problems affecting quality of life**		
Yes	1049	30.6
No	2379	69.4
**Keep working during the pandemic**		
Yes	877	25.6
No	887	25.9
Missing^b^	1664	48.5
**Infected by COVID‐19**		
Yes	36	1.1
No	1727	50.4
Missing^b^	1665	48.6
**Total score**		
	**Mean**	**Standard deviation**
FCV‐19S	16.5875	6.1956
SOC‐3	3.9294	1.3055
CORE‐6D	0.7485	0.1615

^a^ Countries with lower frequencies were grouped.

^b^ The survey was conducted in two steps and this question was not in the first step.

As regard to the general health of the participants, the majority (77.1%) highlighted having a good to very good self‐reported health status during the pandemic. Results also indicate that 16.1% of the subjects were fairly to highly financially affected by the pandemic. Furthermore, Table 1 shows a mean score of 3.93 (*SD* ± 1.31) for SOC‐3 and 0.75 (*SD* ± 0.16) for CORE‐6D.

As noted earlier, several psychometric tests were conducted in this sample of 3428 participants to ascertain the Canadian French fear of COVID‐19 scale's reliability and validity, including internal consistency, factor analysis, construct analysis, concurrent validity, and Rasch analysis.

Table 2 presents the descriptive statistics and corrected item‐total correlations for all seven items of the Canadian French FCV‐19S. Mean item scores ranged from 1.77 to 3.16 (min–max: 1–5). The mean score of the total FCV‐19S was 16.59 (min–max: 7–35) (SD = 6.196). Corrected item‐total correlations were *ρ* = 0.629 or greater, well beyond the value of 0.4 recommended by Wang et al. (2007).

**TABLE 2:** Descriptive statistics, corrected item‐total correlation and PCA of the items of the Canadian French fear of COVID‐19 scale (*n* = 3428)

Items	Mean	Confidence interval (95%)	Corrected item‐total correlation^a^	Variance	Skewness	Kurtosis	PCA factor loading
FearCOVID1	3.16	3.12–3.20	.629	1.346	−0.304	−0.770	0.710
FearCOVID2	2.79	2.75–2.83	.692	1.284	0.007	−0.886	0.769
FearCOVID3	1.90	1.86–1.93	.730	1.022	1.022	0.467	0.819
FearCOVID4	2.52	2.48–2.56	.740	1.497	0.345	−0.867	0.811
FearCOVID5	2.50	2.46–2.54	.741	1.386	0.244	−0.964	0.818
FearCOVID6	1.77	1.74–1.80	.710	0.975	1.263	1.061	0.805
FearCOVID7	1.95	1.92–1.99	.764	1.170	0.929	−0.033	0.847

^a^ Alpha > .4 is considered acceptable.

The present study also analyzed the distribution of the seven FCV‐19S items. Two items (3 and 6), as shown in Table 2, were asymmetrically distributed. As for asymmetry and Kurtosis, two items, which do not fall in the range of –1 and +1, were distributed in a nonnormal way (Tabachnick & Fidell, 2007). More specifically, using the Shapiro–Wilk normality test, all items were distributed in a nonnormal way (*p* < .01). Moreover, the Canadian French FCV‐19S appeared to have a unidimensional structure. All seven items were specified as indicators of a single factor with loading values ranging from 0.71 to 0.847.

Table 3 shows that intercorrelations between the FCV‐19S items ranged from *ρ* = 0.537 to *ρ* = 0.769. A positive relationship between all items (Table 3) was observed (min = 0.359, max = 0.769, all items are statistically significant for *p* ≤ .01). These results indicate that the Canadian French FCV‐19S presented a good fit to the data.

**TABLE 3:** Item–total correlations and intracorrelations

	FearCOVID1	FearCOVID2	FearCOVID3	FearCOVID4	FearCOVID5	FearCOVID6	FearCOVID7	Total score
FearCOVID1	−	−	−	−	−	−	−	.734*
FearCOVID2	.630*	−	−	−	−	−	−	.781*
FearCOVID3	.411*	.537*	−	−	−	−	−	.801*
FearCOVID4	.672*	.562*	.562*	−	−	−	−	.823*
FearCOVID5	.523*	.611*	.588*	.602*	−	−	−	.821*
FearCOVID6	.359*	.469*	.730*	.543*	.586*	−	−	.784*
FearCOVID7	.439*	.520*	.712*	.598*	.647*	.769*	−	.831*

* Correlation is significant at the .01 level (two‐tailed), *n* = 3428.

The psychometric testing has also shown that the FCV‐19S has a high internal consistency with a Cronbach's alpha of .903 for the seven‐item scale, which could not be improved by removing any items.

Concurrent validity was supported by examining the association with the Sense of Coherence scale 3‐item (SOC‐3) and the CORE‐6D. The FCV‐19S demonstrated good concurrent validity. There was a moderate negative relationship between the FCV‐19S and the SOC‐3 scale (*r* = –.233, *p* < .001), such that those who had higher fear of COVID‐19 scores reported lower overall ability to cope with the situation caused by the COVID‐19. FCV‐19S showed a good negative relationship with CORE‐6D (*r* = –.410, *p* < .001) indicating that those with higher fear scores reported higher levels of emotional and physical distress.

The Rasch analysis is reported in Table 4. The inlier sensitive fit mean square and the outlier sensitive fit mean square (respectively Infit MnSq and Outfit MnSq) represent the range out of which the item does not function the way it is supposed to function. For the Infit MnSq, all items show satisfactory values comprised between 0.78 and 1.25. For the Outfit MnSq, the same appreciation is done, and values are comprised between 0.71 and 1.24.

**TABLE 4:** Psychometric properties (*n* = 3428)

Item	Model SE	Infit MnSq	Outfit MnSq	Difficulty
FearCOVID1	0.17	0.95	0.89	−1.63
FearCOVID2	0.16	1.24	1.24	−0.69
FearCOVID3	0.19	0.84	0.75	1.14
FearCOVID4	0.16	1.25	1.12	−0.37
FearCOVID5	0.16	1.04	1.03	−0.53
FearCOVID6	0.2	0.78	0.71	1.51
FearCOVID7	0.18	0.97	0.86	0.58

The person separation index and the person separation reliability are, respectively, about 2.44 and 0.86. These values are greater than the minimal reference values (>2 and >0.7, respectively). The item separation index and the item separation reliability show satisfactory results and are equal to 5.64 and 0.97, respectively (see Table 5), which is also satisfactory.

**TABLE 5:** Other psychometric properties (*n* = 3428)

Psychometric testing	Value	Suggested cut‐off
Standard error of measurement (mean)	0.17	The smaller the better
Item separation reliability from Rasch	0.97	>0.7
Item separation index from Rasch	5.64	>2
Person separation reliability from Rasch	0.86	>0.7
Person separation index from Rasch	2.44	>2

Additional analysis also indicates that age was negatively correlated with the FCV‐19S but was not significant (*r* = –.016, *p* = .170). However, results have shown a significant positive correlation between gender and the pattern of FCV‐19S (*r* = .085, *p* < .01).

## 4 DISCUSSION

The present study detailed the process of translating the fear of COVID‐19 Scale (FCV‐19S) into Canadian French with transcultural validation as well as to present the psychometric properties of the questionnaire. Results have shown that the Canadian French FCV‐19S (CF‐FCV‐19S) is an easy to read and understand questionnaire. The study findings support that the CF‐FCV‐19S is a reliable self‐report measure with good psychometric properties in the population residing in the province of Quebec. Results indicated a stable unidimensional structure of the CF‐FCV‐19S, confirming the findings of the original validation study in Farsi (Ahorsu et al., 2020) and in English (Winter et al., 2020). Moreover, the overall score of the summed‐up items scores can indicate the severity of the fear of COVID‐19, as it was demonstrated by Ahorsu et al. (2020). More specifically, the sum of the item scores represents the severity of the perceived fear (i.e., a higher score indicates a more severe state of fear). Moreover, in line with the findings of Ahorsu et al. (2020) and Soraci et al. (2020), our study demonstrated that the responses pattern of CF‐FCV‐19S was not affected by the age of the respondents. Therefore, it can be concluded that the CF‐FCV‐19S could offer a good insight into the fear of COVID‐19 to address the psychological problems emanating from COVID‐19 in adult people of all ages at risk and advise them accordingly. However, our findings diverge from those of Ahosru et al. (2020) and indicated that the gender significantly affected the responses to the FCV scale. So, men completed differently from women the questions of the fear of COVID‐19 and this may be because women experience a higher prevalence of fear‐related emotional issues than men (Villarreal‐Zegarra & Bernabe‐Ortiz, 2020).

Psychometric analyses showed that the CF‐FCV‐19S has a good internal reliability and consistency when assessed by Cronbach's alpha (.903). This is in line with previous internal consistency findings from other countries (Iversen et al., 2021; Ahorsu et al., 2020; Alyami et al., 2021; Tzur Bitan et al., 2020; Reznik et al., 2021; Sakib et al., 2020; Satici et al., 2021; Soraci et al., 2020; Tsipropoulou et al., 2020; Winter et al., 2020, Huarcaya‐Victoria et al., 2022; Pang et al., 2020) reporting a Cronbach's alpha between .82 and .89. As indicated by the results (Table 3), each individual corrected item correlated >.70 with the total score, indicating satisfactory internal consistency (Everitt & Skrondal, 2010).

In previous validation studies, the FCV‐19S total score showed good concurrent validity with other constructs measuring, for example, anxiety, depression, and distress (Iversen et al., 2021; Soares et al., 2021; Ahorsu et al., 2020; Alyami et al., 2021; Tzur Bitan et al., 2020; Reznik et al., 2021; Sakib et al., 2020; Satici et al., 2021; Soraci et al., 2020; Tsipropoulou et al., 2020; Winter et al., 2020; Huarcaya‐Victoria et al., 2022; Pang et al., 2020; Chang et al., 2020). Our results were in line with these studies and confirmed good concurrent validity as the CF‐FCV‐19 total score was significantly correlated with the scales measuring emotional and physical distress (CORE‐6D) and the ability to cope with life situation (SOC‐3). So, people with severe fear of COVID‐19 may be more affected by stress and anxiety with limited ability to deal with the stress caused by COVID‐19 infection, thereby increasing the stress levels for individuals and health care workers. The stress generated in turn may worsen their well‐being (CDC, 2020c) and make them more prone to disease (Morey et al., 2015). According to previous studies, it has been stated that the health of individuals can be affected by negative psychological states such as anxiety during periods of infectious epidemics (Duncan et al., 2009; Pappas et al., 2009; Ropeik, 2004). Therefore, the CF‐FCV‐19S could help the population better understand the emotional factors associated with pandemics and subsequently help reduce anxiety and stress (CDC, 2020a, 2020b).

## 5 STRENGTHS AND LIMITATIONS

Psychometric tests of the CF‐FCV‐19S demonstrated that the CF‐FCV‐19S is a reliable and valid tool to assess the severity of fear of COVID‐19 in Quebec adults of all ages. The study was also conducted in a quite representative and large sample of the general population in Quebec. However, the present study has certain limitations. First, since the assessment of the concept of fear is subjectively carried out; however, the social desirability factors that may influence participants' responses must be considered. To mitigate this bias, an online survey without human interaction can be performed. Second, although self‐report data are a valuable information resource for constructs, self‐report bias must nonetheless be taken into account (Althubaiti, 2016). Third, in surveys, there is always a possibility of self‐selection bias (Bethlehem, 2010), which may indicate an overrepresentation of specific subgroups within the general population. In addition, the sample in the current study did not include adolescents and children. Finally, participants were from the general population residing in the province of Quebec and no formal diagnosis on mood disorders was obtained (e.g., anxiety). Therefore, the sensitivity and specificity of the scale cannot be examined. Future studies should thus evaluate if individuals with underlying medical conditions associated with a higher risk of death from COVID‐19 (e.g., diabetes, hypertension, coronary heart disease, preexisting respiratory conditions) may experience increased levels of COVID‐19 fear.

## 6 CONCLUSION

The translation and transcultural validation of the CF‐FCV‐19S led to an easy‐to‐read and ‐understand questionnaire. In addition, the psychometric testing of the CF‐FCV‐19S demonstrated that the instrument is a unidimensional reliable and valid tool for assessing the severity of fear of COVID‐19 among adults of all ages residing in the province of Quebec.

### PEER REVIEW

The peer review history for this article is available at https://publons.com/publon/10.1002/brb3.2550.

## CONFLICT OF INTEREST

The authors declare that they have no conflict of interest.

## INFORMED CONSENT

All participants gave their informant consent. The study was approved by the Ethics Committee of the CIUSSS de l'Est de l’Île de Montréal.

## Data Availability

Data will be available upon reasonable request to the corresponding author.

## Canadian French version of the fear of COVID‐19 scale

### Échelle de peur de la COVID‐19

Veuillez répondre à chaque point en sélectionnant l'une des cinq (5) réponses qui reflète comment vous vous sentez, pensez ou agissez face à la COVID‐19.

**Table:**

	Fortement en désaccord	En désaccord	Neutre	En accord	Fortement en accord
1. J'ai très peur de la COVID‐19 2. Cela me met mal à l'aise de penser à la COVID‐19 3. Mes mains deviennent moites quand je pense à la COVID‐19 4. J'ai peur de perdre la vie à cause de la COVID‐19 5. Quand je regarde les nouvelles et les histoires sur la COVID‐19 sur les réseaux sociaux, je deviens nerveux ou anxieux 6. Je ne peux pas dormir parce que je suis inquiet d'attraper la COVID‐19 7. Mon cœur bat la chamade ou palpite quand j'imagine attraper la COVID‐19					
